# Exploration of Prognostic Biomarkers for Lung Adenocarcinoma Through Bioinformatics Analysis

**DOI:** 10.3389/fgene.2021.647521

**Published:** 2021-04-22

**Authors:** Zhengliang Tu, Xiangfeng He, Liping Zeng, Di Meng, Runzhou Zhuang, Jiangang Zhao, Wanrong Dai

**Affiliations:** ^1^Department of Thoracic Surgery, The First Affiliated Hospital, Zhejiang University School of Medicine, Hangzhou, China; ^2^Department of Thoracic Surgery, Zhuji People’s Hospital, Zhuji, China; ^3^Department of Pharmacy, The First Affiliated Hospital, College of Medicine, Hangzhou, China

**Keywords:** lung adenocarcinoma, prognosis, biomarker, gene signature, TCGA

## Abstract

With the development of computer technology, screening cancer biomarkers based on public databases has become a common research method. Here, an eight-gene prognostic model, which could be used to judge the prognosis of patients with lung adenocarcinoma (LUAD), was developed through bioinformatics methods. This study firstly used several gene datasets from GEO database to mine differentially expressed genes (DEGs) in LUAD tissue and healthy tissue via joint analysis. Later, enrichment analysis for the DEGs was performed, and it was found that the DEGs were mainly activated in pathways involved in extracellular matrix, cell adhesion, and leukocyte migration. Afterward, a TCGA cohort was used to perform univariate Cox, least absolute shrinkage and selection operator method, and multivariate Cox regression analyses for the DEGs, and a prognostic model consisting of eight genes (GPX3, TCN1, ASPM, PCP4, CAV2, S100P, COL1A1, and SPOK2) was established. Receiver operation characteristic (ROC) curve was then used to substantiate the diagnostic efficacy of the prognostic model. The survival significance of signature genes was verified through the GEPIA database, and the results exhibited that the risk coefficients of the eight genes were basically congruous with the effects of these genes on the prognosis in the GEPIA database, which suggested that the results were accurate. Finally, combined with clinical characteristics of patients, the diagnostic independence of the prognostic model was further validated through univariate and multivariate regression, and the results indicated that the model had independent prognostic value. The overall finding of the study manifested that the eight-gene prognostic model is closely related to the prognosis of LUAD patients, and can be used as an independent prognostic indicator. Additionally, the prognostic model in this study can help doctors make a better diagnosis in treatment and ultimately benefit LUAD patients.

## Introduction

Lung adenocarcinoma (LUAD) is a common type of non-small cell lung carcinoma (NSCLC), with an incidence of 40% of all lung cancers (Jiang et al., 2020). Studies manifested that long-term smoking, air pollution, and familial inheritance all contribute to the pathogenesis of LUAD (Hackshaw et al., 1997; Byun et al., 2018; Tseng et al., 2019). At present, treatment for LUAD mainly includes surgical treatment and drug treatment. Surgical treatment is mainly to completely eradicate tumor by performing surgery on patients to remove cancer tissue (Ikehara et al., 2012). Drug treatment targets cancer cell metabolic characteristics or transcription and translation and cell structure characteristics, so as to treat LUAD through specific pathways targeting cancer cells (Saito et al., 2016; Scafoglio et al., 2018; Skoulidis et al., 2018). For instance, TKI can restrain the growth of tumor cells by repressing tyrosine kinase activity (Yaish et al., 1988). Besides, Ado-trastuzumab emtansine can hinder tumor progression by targeting and suppressing HER-2 (Li et al., 2018). In addition to targeted drugs that kill tumor cells, researchers have recently tried to treat LUAD patients through immunotherapy (Saito et al., 2018). Immunotherapy mainly blocks the immunosuppression of cancer cells or promotes the activity of immune cells by using drugs, and treats patients by activating their immune system. In the past 10 years, the application of the above-mentioned various drugs has greatly improved the survival time of patients with advanced lung cancer. However, due to the characteristics of early metastasis of lung cancer, the survival improvement of patients with targeted drugs still encounters a bottleneck (Auperin et al., 2010; Travis et al., 2013).

Current research found that the mortality rate of lung cancer is related to its diagnosis time, which indicates that earlier treatment can greatly elevate the survival rate of patients if cancer diagnosed in early stages (Yu et al., 2010). Therefore, numerous studies have tried to find biomarkers that can be used to determine whether a patient has cancer by comparing the physiological conditions between patients and healthy people. For example, Scafoglio et al. (2018) found that the SGLT2 gene can be used as a biomarker of early LUAD, which can distinguish lung nodules and early cancer, and improve the survival rate of cancer patients. In summary, lung cancer is a modern disease with extremely high morbidity and mortality, and early diagnosis as well as classification of lung cancer is of great value to the prognosis of patients (Yu et al., 2010).

With the pervasion of second-generation sequencing, it is currently a prevalent cancer research method that analyzing high-throughput expression data of cancer patients through bioinformatics methods. A study constructed a tumor classifier for early tumor diagnosis with machine learning algorithms (Jiao et al., 2020). Another study established a risk prognostic model to conduct risk prediction for patients, beneficial for clinicians to make personalized diagnosis and treatment (Zhang et al., 2013). Currently, feature selection, multi-chip joint analysis, Gene Ontology (GO) and Kyoto Encyclopedia of Genes and Genomes (KEGG) enrichment analyses, Cox regression analysis are all common methods for bioinformatics analysis. Feature selection method screens out the genes that matter the most on disease to contribute to follow-up research, and CHNMF, HSNMF, and DSTPCA are all the feature selection algorithms proposed in recent years (Hu et al., 2019; Wang C. et al., 2020; Yu et al., 2020). Multi-chip joint analysis can integrate various datasets. Gao et al. (2018) screened differentially expressed genes (DEGs) from multiple expression profiles of bladder cancer through multi-chip joint analysis, and finally identified hub genes related to bladder cancer pathogenesis from the protein–protein interaction (PPI) network constructed by the identified DEGs. As for enrichment analysis, Song et al. (2020) applied this method to elucidate the function of DEGs in hepatocellular carcinoma. Additionally, in terms of regression analysis, a study established an immune-related prognostic model for hepatocellular carcinoma through regression analysis, and the model can accurately and effectively determine the outcomes of patients (Chen et al., 2020).

In the present study, joint differential analysis was firstly performed to screen out DEGs in LUAD from three independent GEO datasets. Then, following regression analyses in TCGA-LUAD dataset, including univariate Cox regression, least absolute shrinkage and selection operator method (LASSO) regression, and multivariate Cox regression, a prognostic model was established. The model efficacy was sequentially validated with an independent validation cohort from GEO, and the prognostic value of each gene in the model was verified on the GEPIA database. Finally, the independence of the model was analyzed. The achievement of the study is conducive to the early diagnosis of lung cancer and drug development.

## Materials and Methods

### Raw Data Preparing

First of all, mRNA profiles along with associated clinical characteristics (Normal: 59, Tumor: 535) (Supplementary Table 1) were obtained from TCGA-LUAD^1^ on May 13, 2020. GSE31210, GSE32665, GSE32863, GSE43458, and GSE72094 datasets were downloaded from GEO database^2^. All datasets met the following inclusion criteria: (1) employed tissue samples were collected from human LUAD and corresponding adjacent or normal tissue; (2) at least 10 samples in total were included in each dataset. Information for all included datasets was detailed in Table 1. For analysis, downloaded data were split into three data cohorts: study cohort (GSE43458, GSE32863, and GSE32665), training cohort (TCGA-LUAD), and validation cohort (GSE31210 and GSE72094) (Supplementary Tables 2, 3).

**TABLE 1:** The gene expression profiles and data characteristics.

Data set	Data type	Platform	Normal	Tumor	Follow-up	Cohort
GSE32665	mRNA	GPL6102	92	87	No	Study
GSE32863	mRNA	GPL6884	58	58	No	Study
GSE43458	mRNA	GPL6244	30	80	No	Study
TCGA-LUAD	mRNA	Illumina	59	535	Yes	Training
GSE31210	mRNA	GPL570	20	226	Yes	Validation
GSE72094	mRNA	GPL15048	0	442	Yes	Validation

### Gene Expression Data Preprocessing

Firstly, the GEO datasets were annotated in accordance with platform annotation files, and the probe IDs were transformed into gene symbols. Probes without matching gene symbols were then removed. The KNN (k-nearest neighbor) method (Troyanskaya et al., 2001) was used to estimate the missing values in the gene expression matrix with the impute.knn function in the R package *impute*, and k value adopted the default value of 10. All gene expression values were log-normalized. Then, the limma package (Ritchie et al., 2015) was used to normalize the transcriptome data. The mean of RNA expression level was accepted in case of duplicates.

### Joint Analysis of Multiple Datasets

Differential expression analysis was performed by R package limma in GSE43458, GSE32863, and GSE32665 datasets. Then, DEGs determined in the three sets were integrated using the RobustRankAggreg package (Kolde et al., 2012). Robust Rank Aggregation (RRA) is a prevalent method of data integration in high-throughput data analysis. Statistical significance was set at | log2FC| ≥ 1.5 and adjusted *p* < 0.05.

### Functional Enrichment Analysis

Gene Ontology and KEGG enrichment analyses were performed by ClusterProfiler package (Yu et al., 2012) to further probe the biological mechanisms of DEGs. The *p*-value here was corrected by calculating the false discovery rate (FDR), and pathways were considered to be significantly activated when FDR < 0.05. According to package instructions, the top 10 pronouncedly enriched biological pathways and biological processes were visualized.

### Identification and Validation of Prognostic Gene Signature

Cox regression, a general method to establish a prognostic risk model, takes survival outcome and survival time as dependent variables to analyze the impact of different variables on survival (Fisher and Lin, 1999). The DEGs which were remarkably associated with overall survival (OS) in TCGA-LUAD cohort were selected through univariate Cox regression analysis (Cox’s proportional hazard regression analysis, PHR analysis) (*p* < 0.05). A LASSO regression model was developed with identified OS-associated genes by using the glmnet package, and the most informative prognostic mRNA biomarkers for OS were distinguished. A multivariate Cox regression model (backward stepwise) was employed to construct the final prognostic model on the basis of screened prognostic mRNA biomarkers.

Risk score was computed with the following equation:

(1) $$\text{Riskscore}=\sum_{i=1}^{n}\left({\text{Coef}}_{i}\times x_{i}\right)$$

where Coef_*i*_ represents the coefficient of each signature gene, and *x*_*i*_ represents the relative expression level of each signature gene.

All samples in TCGA-LUAD were given a risk score and were separated into high- and low-risk groups with the median risk score as a cut-off value. The OS between patients with low and high risks was compared via Kaplan–Meier survival analysis. The sensitivity and specificity of the model in prognosis prediction were inspected through receiver operation characteristic (ROC) analysis (Obuchowski and Bullen, 2018), and prognostic accuracy was analyzed by area under curve (AUC) values. The prognostic model was then validated in two independent LUAD cohorts (GSE31210 and GSE72094).

### Gene Expression Profiling Interactive Analysis (GEPIA)

Prognostic effect of each signature gene of the model was verified by Gene Expression Profiling Interactive Analysis (GEPIA) database^3^. GEPIA is a web server that analyzes gene expression data of a large number of samples in TCGA and Genotype-Tissue Expression (GTEx).

### Independence Analysis of Prognostic Model

Independence of the prognostic model was authenticated by univariate and multivariate Cox regression. Concisely, the model-based risk score and traditional clinical characteristics (age, gender, pathologic_T stage, and clinical stage) for LUAD patients were employed as independent variables while OS was taken as a dependent variable. Statistical significance was assumed at *p* < 0.05.

### Construction of Nomogram

Age, gender, clinical stage, pathologic_T stage, and risk score were used to create a nomogram that could predict the likelihood of OS of LUAD patients. The survival and the rms packages were used to establish the nomogram.

## Results

### Differences in mRNA Expression Between LUAD and Normal Tissue

**FIGURE 1:**
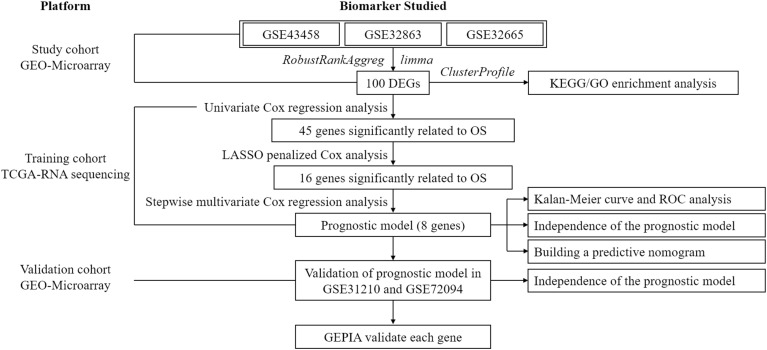
The flowchart shows the overall analytical process of this study.

**FIGURE 2:**
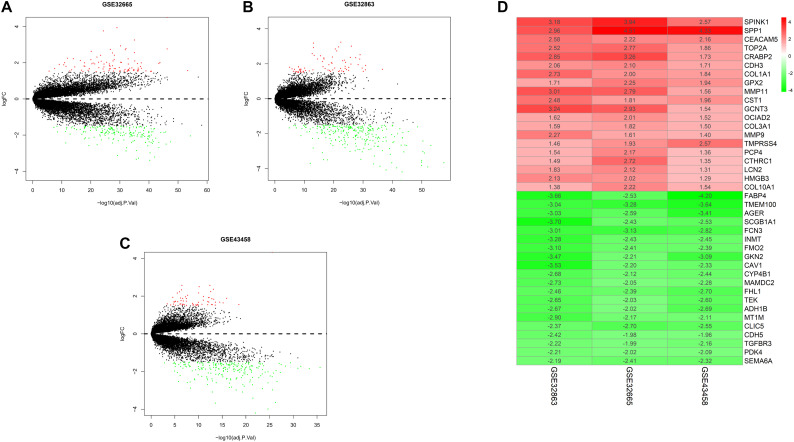
Differential expression gene between normal and tumor tissue in three datasets, and heatmap of DEGs. **(A)** Volcano plot of GSE32665. The red points represent up-regulated genes, the green points represent down-regulated genes, and the black points represent genes without significant difference; **(B)** volcano plot of GSE32863; **(C)** volcano plot of GSE43458; **(D)** heatmap of top 20 up-regulated and down-regulated DEGs in three datasets.

To exhibit the analytical process more clearly, the flowchart of this study was drawn in Figure 1. Joint analysis was performed on GSE43458, GSE32863, and GSE32665 to analyze the differences in gene expression between cancer tissue and healthy tissue. The results displayed that there were 100 DEGs in the three datasets, among which 38 DEGs were up-regulated, and 62 DEGs were down-regulated (Figures 2A–C). The top 20 up-regulated DEGs and top 20 down-regulated DEGs with the most significant expression difference were listed in Figure 2D.

### Enrichment Analyses of DEGs

**FIGURE 3:**
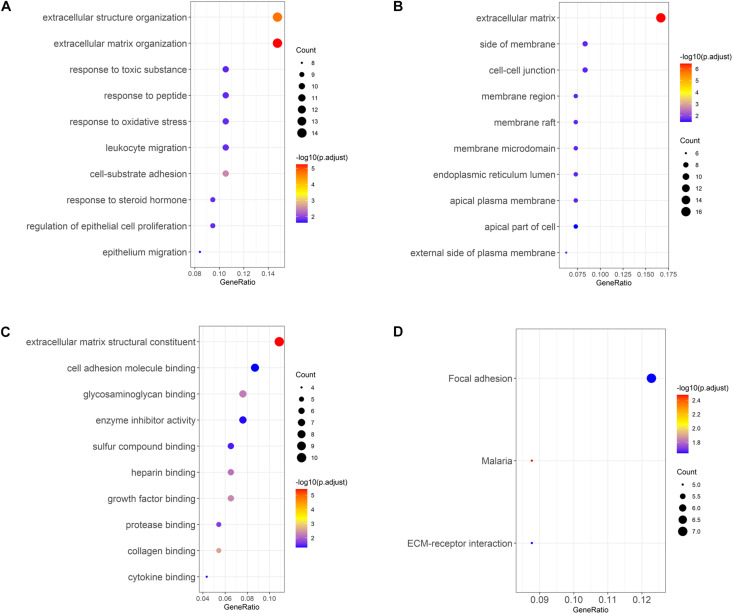
GO and KEGG analyses of LUAD DEGs. **(A–C)** GO enrichment analysis of LUAD DEGs, separated for biological process (BP), cellular component (CC), and molecular function (MF); **(D)** KEGG enrichment analysis of LUAD DEGs.

Gene Ontology enrichment analysis of the selected DEGs revealed that DEGs were mainly enriched in biological processes such as extracellular structure organization, response to toxic substance, leukocyte migration (Figure 3A), cellular components such as cell–cell junction, membrane region, apical plasma membrane (Figure 3B), and molecular functions such as glycosaminoglycan binding, enzyme inhibitor activity, growth factor binding (Figure 3C). In addition, KEGG enrichment analysis was performed, and it was found that DEGs were enriched in pathways involved in focal adhesion and ECM-receptor interactions (Figure 3D). The above results demonstrated that genes differentially expressed in LUAD tissue and normal tissue were mainly activated in the pathways relevant to extracellular matrix, cell adhesion, and leukocyte migration.

### Construction and Verification of LUAD Prognostic Model

**FIGURE 4:**
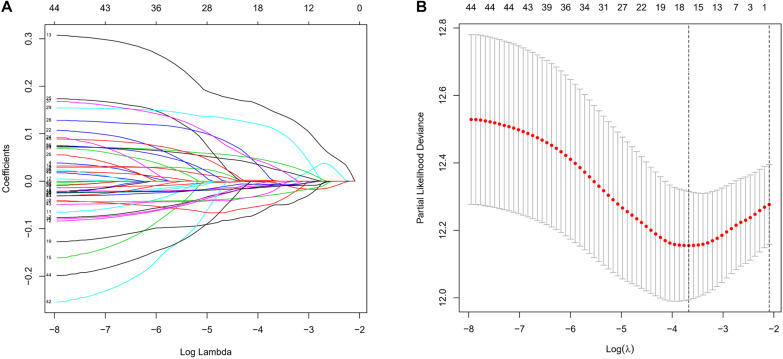
Establishment of prognostic risk model by LASSO regression analysis. **(A)** LASSO coefficient profiles of the 45 prognosis-related genes in TCGA-LUAD. A coefficient profile plot was generated against the log (lambda) sequence; **(B)** selection of the optimal parameter (lambda) in the LASSO model.

**FIGURE 5:**
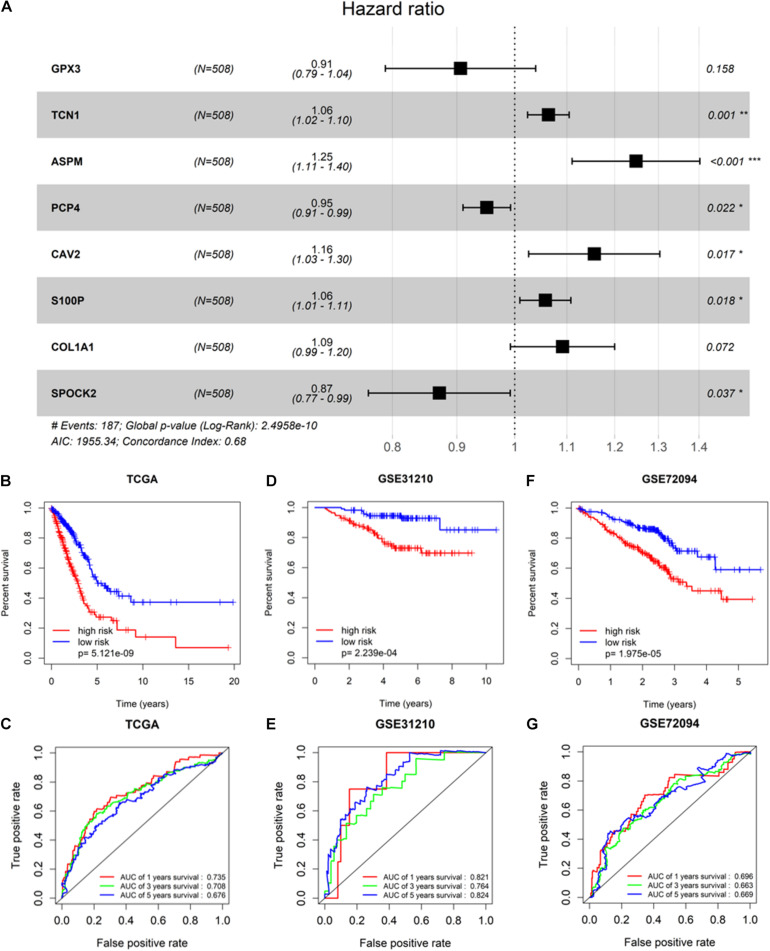
Validation of prognostic risk model. **(A)** Gene signature filtered by multivariate Cox regression analysis; **(B,D,F)** Kaplan–Meier survival curve analysis in TCGA-LUAD cohort, GSE31210, and GSE72094, respectively (patients were grouped by the median risk score); **(C,E,G)** ROC analysis of the sensitivity and specificity of the OS for the eight-gene risk score in TCGA-LUAD, GSE31210, and GSE72094, respectively. * Represents *p-value* <0.05, ** represents *p-value* <0.01, *** represents *p-value* <0.001, and # represents annotation information.

To screen survival-related genes of LUAD, TCGA-LUAD dataset was set as the training cohort. Univariate Cox regression analysis was firstly performed on the 100 DEGs obtained in the differential gene analysis, and 45 DEGs that were notably related to the OS of patients were screened out (Supplementary Table 4). Subsequently, regression coefficients of the 45 DEGs were evaluated by LASSO regression analysis (Figure 4A). It was finally verified through cross-validation that 16 DEGs could achieve a better effect in the model (Figure 4B; Supplementary Table 5). Eventually, multivariate Cox stepwise regression method was used to establish several multivariate regression models. A risk model consisting of 8 DEGs (GPX3, TCN1, ASPM, PCP4, CAV2, S100P, COL1A1, and SPOK2) was at last identified (Figure 5A). GPX3, PCP4, and SPOCK2 were low-risk genes, while TCN1, ASPM, CAV2, S100P, and COL1A1 were high-risk genes.

After the risk model was constructed, the reliability of the model was verified in both the training cohort and validation cohort. Based on the model, patients in the two cohorts were scored, and then divided into high-risk group and low-risk group with the median risk score as the cut-off. The results of survival analysis illustrated that the OS of high-risk patients in the three datasets (TCGA-LUAD, GSE31210, and GSE72094) was lower than that of low-risk patients, indicating that high-risk patients showed a markedly worse prognosis (Figures 5B,D,F). ROC analysis revealed that AUC values of the patients in the training cohort for 1-, 3-, and 5-year survival were 0.735, 0.708, and 0.676, respectively, indicating the good diagnostic efficacy of the model (Figure 5C). While for the validation cohort (GSE31210 and GSE72094), the AUC values were all greater than 0.65, suggesting that the risk model had a certain universality in determining the OS of LUAD patients (Figures 5E,G). The above results indicated that the constructed risk model had good diagnostic performance and could be used to predict the prognostic risk of LUAD patients.

### GEPIA Validates Prognostic Feature Genes

**FIGURE 6:**
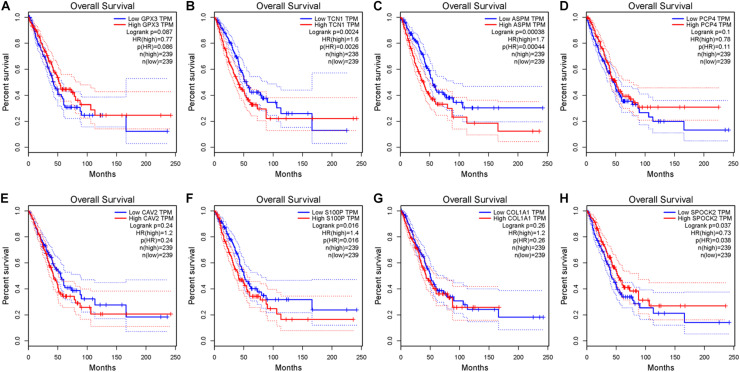
Survival analysis of eight signature genes in GEPIA website. **(A–H)** Survival analysis of GPX3, TCN1, ASPM, PCP4, CAV2, S100P, COL1A1, and SPOK2 in GEPIA database.

After the accuracy of the risk model was verified, the relationship between signature gene expression and patient’s survival was also verified through the GEPIA database. The results displayed that patients with high expression of TCN1, ASPM, and S100P had pronouncedly shorter OS, while patients with high expression of SPOCK2 had dramatically longer OS (Figures 6A–H). These results were congruous with the results of multivariate Cox regression analysis. These prognostic signature genes could be used to determine the survival of patients.

### Verification of Independence of the LUAD Prognostic Risk Model and Establishment of a Prognostic Nomogram

**FIGURE 7:**
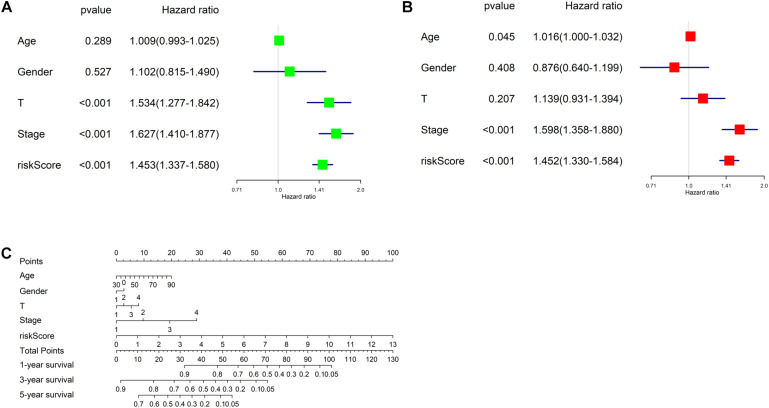
Independent analysis of risk model and construction of nomogram. **(A)** Univariate Cox regression analysis in TCGA-LUAD cohort; **(B)** multivariate Cox regression analysis in TCGA-LUAD cohort; **(C)** the nomogram for predicting OS.

Univariate and multivariate regression analyses were performed in TCGA-LUAD dataset combined with traditional clinical indicators (age, gender, pathologic_T stage, and clinical stage) and risk score. Results exhibited that the model-based risk score was remarkably associated with the OS of patients (Figures 7A,B), which manifested that the risk score could be used as an independent indicator of prognosis of patients.

Afterward, a prognostic nomogram was established based on the traditional clinical indicators of patients and the risk score (Figure 7C). The nomogram we established could integrate clinical indicators and the risk score to assess patient’s survival.

## Discussion

Lung cancer is a common disease in modern times, and LUAD is a common type of lung cancer (Jemal et al., 2007). LUAD is a heterogeneous disease, and even in patients with similar clinical symptoms they may not have a close OS (Hua et al., 2020). Besides, there still exists differences in patients receiving the same treatment, and some can be cured while some will relapse (Chen et al., 2007). Based on the abovementioned, it is believed that there is a bottleneck in determining the prognosis of LUAD patients through traditional clinical indicators. Thus, development of more methods for determining the prognostic risk of LUAD patients is in need. Mining biomarkers that affect patient’s prognosis through public databases is in common use (Zhang et al., 2019). This method can screen out signature genes related to the prognosis of LUAD patients with no need for a large quantity of sample collection, sequencing experiments, and costs.

In this study, data in GSE43458, GSE32863, and GSE32665 datasets from GEO database were extracted to compare gene expression between LUAD tumor tissue and healthy tissue, and 100 DEGs were screened out. The results of KEGG and GO enrichment analyses revealed that these DEGs were mainly enriched in pathways related to extracellular matrix, cell adhesion, and leukocyte migration. The extracellular matrix is an important part of the tumor microenvironment and a commonly affected pathway in tumor cells (Venning et al., 2015). Cell adhesion is also a pathway closely related to tumor progression. A study (Laubli and Borsig, 2019) found that the decrease of surface adhesion proteins in tumor cells can lead to weakened cell adhesion ability, ultimately causing tumor migration and invasion. Besides, leukocyte migration is a pivotal pathway related to anti-tumor immunity. The migration of leukocytes to tumor tissue can stimulate inflammation and kill cancer cells. A study (Cochran et al., 1976) found that the serum of patients can restrain the migration of cancer cells.

Simultaneously, an eight-gene prognostic model was further constructed through univariate Cox analysis, LASSO, and multivariate Cox regression analysis based on TCGA-LUAD dataset and OS of patients. The eight genes were GPX3, TCN1, ASPM, PCP4, CAV2, S100P, COL1A1, and SPOK2. Among them, GPX3, PCP4, and SPOCK2 were low-risk genes, while TCN1, ASPM, CAV2, S100P, and COL1A1 were high-risk genes. GPX3 is a tumor suppressor gene that takes an important part in balancing reactive oxygen species (ROS) in colitis, thereby inhibiting cancer progression (Barrett et al., 2013). PCP4 is a protein that promotes the differentiation of nerve cells, and research suggested that PCP4/PEP19 can promote the migration and invasion of breast cancer (Honjo et al., 2018). However, the role of PCP4 alone in cancer remains an open issue, and the changes of PCP4/PEP19 in LUAD have not yet been explored. This study believed that PCP4 in LUAD was beneficial to the prognosis of patients. SPOCK2 is the core protein of proteoglycan Testican-2/SPOCK2, and Testican-2/SPOCK2 is an interferon-induced proteoglycan that plays an antiviral effect *in vivo* (Ahn et al., 2019). The overall role of SPOCK2 in LUAD has not yet been fully defined, but this study found that the expression of SPOCK2 was beneficial to the survival of LUAD patients. TCN1 is a vitamin B12 binding protein that can regulate the homeostasis of cobalamin *in vivo*. Research suggested that TCN1 is negatively related to patient’s prognosis, and it can promote tumor migration, invasion, and reduce the chemotherapy sensitivity of cancer cells (Liu et al., 2020). ASPM is a traditional oncogene. A study believed that ASPM is highly expressed in cancer tissue of LUAD patients and is closely related to the occurrence of lung cancer, with prognosis significance (Wang J. et al., 2020). CAV2 is an oncogene that can promote the growth of renal cell carcinoma through the EGFR/PI3K/Akt pathway (Liu et al., 2018). S100P is a member of the S100 protein family. The S100 protein family is widely involved in various stages of occurrence and progression of tumor. Research suggested that S100P can stimulate the progression of a variety of cancers and acts as an oncogene (Wang et al., 2018). COL1A1 is considered to have a cancer-promoting effect. It is found that COL1A1 can promote the occurrence of lung cancer (Bibaki et al., 2018).

Following the establishment of the risk model, GEPIA database was used to verify the relationship between these signature genes and patient’s prognosis. The results of GEPIA were consistent with the finding of this study, suggesting that the prognostic signature genes selected in this study were accurate. Subsequently, the independence of the risk model was validated using univariate and multivariate regression combined with clinical characteristics. The results denoted that the risk model could be used as an independent prognostic factor. In addition, combined with clinical information, a prognostic nomogram was established to guide clinical diagnosis.

## Conclusion

In conclusion, the eight prognostic signature genes identified in this study were prominently related to the OS of LUAD patients. Determination of the prognosis of LUAD patients based on the eight-gene risk model is beneficial for clinicians to make the correct diagnosis, to discover the prognostic risk of patients in advance, and to improve the survival of patients. Although the above analyses fully proved that the eight genes could be used as prognostic signature genes to determine the survival of patients, this study is a pure bioinformatics study that only used data in public databases to screen prognostic biomarkers without clinical trials. To prove the clinical application value of the eight genes, further clinical trials are still needed.

## Data Availability Statement

The data used to support the findings of this study are included within the article. The data and materials in the current study are available from the corresponding author on reasonable request.

## Author Contributions

All authors contributed to data analysis, drafting, and revising the article, gave final approval of the version to be published, and agreed to be accountable for all aspects of the work.

## Conflict of Interest

The authors declare that the research was conducted in the absence of any commercial or financial relationships that could be construed as a potential conflict of interest.

## Footnotes

1 https://portal.gdc.cancer.gov/

2 https://www.ncbi.nlm.nih.gov/geo/

3 http://gepia.cancer-pku.cn/

## Supplementary Material

Supplementary Table 1 Clinical information of patients in TCGA-LUAD dataset.

Supplementary Table 2 Clinical information of patients in GSE31210 dataset.

Supplementary Table 3 Clinical information of patients in GSE72094 dataset.

Supplementary Table 4 Results of univariate Cox regression analysis.

Supplementary Table 5 Results of LASSO regression analysis.
